# Big data hurdles in precision medicine and precision public health

**DOI:** 10.1186/s12911-018-0719-2

**Published:** 2018-12-29

**Authors:** Mattia Prosperi, Jae S. Min, Jiang Bian, François Modave

**Affiliations:** 1 Department of Epidemiology, College of Medicine & College of Public Health and Health Professions, University of Florida, Gainesville, FL 32610 USA; 2 Department of Health Outcomes and Biomedical Informatics, College of Medicine, University of Florida, Gainesville, FL 32610 USA; 3 Center for Health Outcomes and Informatics Research, Loyola University Chicago, Maywood, IL 60153 USA

## Abstract

**Background:**

Nowadays, trendy research in biomedical sciences juxtaposes the term ‘precision’ to medicine and public health with companion words like big data, data science, and deep learning. Technological advancements permit the collection and merging of large heterogeneous datasets from different sources, from genome sequences to social media posts or from electronic health records to wearables. Additionally, complex algorithms supported by high-performance computing allow one to transform these large datasets into knowledge. Despite such progress, many barriers still exist against achieving precision medicine and precision public health interventions for the benefit of the individual and the population.

**Main body:**

The present work focuses on analyzing both the technical and societal hurdles related to the development of prediction models of health risks, diagnoses and outcomes from integrated biomedical databases. Methodological challenges that need to be addressed include improving semantics of study designs: medical record data are inherently biased, and even the most advanced deep learning’s denoising autoencoders cannot overcome the bias if not handled a priori by design. Societal challenges to face include evaluation of ethically actionable risk factors at the individual and population level; for instance, usage of gender, race, or ethnicity as risk modifiers, not as biological variables, could be replaced by modifiable environmental proxies such as lifestyle and dietary habits, household income, or access to educational resources.

**Conclusions:**

Data science for precision medicine and public health warrants an informatics-oriented formalization of the study design and interoperability throughout all levels of the knowledge inference process, from the research semantics, to model development, and ultimately to implementation.

## Background

The United States White House initiative on precision medicine stated that its mission is *“to enable a new era of medicine through research, technology, and policies that empower patients, researchers, and providers to work together toward development of individualized care”* [1]. Our ability to store data now largely surpasses our ability to effectively and efficiently learn from them and to develop actionable knowledge that leads to improvements in health outcomes. Precision medicine sprouts from big data and is the manifest evidence of such a dramatic change in scientific thinking. However, from its inception, precision medicine has been beleaguered with technical and sociopolitical challenges [2].

### What is precision medicine?

The National Institutes of Health (NIH) defines precision medicine as the *“approach for disease treatment and prevention that takes into account individual variability in genes, environment, and lifestyle for each person”* [3]. The emphasis is placed on tailored prevention, diagnosis and treatment for each individual based on genetics, epigenetics, and other lifestyle considerations. The terms ‘personalized,’ ‘stratified’ and ‘individualized’ medicine have been often used interchangeably, but superseded lately by ‘precision’ [4]. Precision has been preferred *“to emphasize the new aspects of this field, which is being driven by new diagnostics and therapeutics”* [5]. Nonetheless, the debate on terms and definitions is still open [6].

A classic example of precision medicine is the customization of disease treatment for a single individual. In the old paradigm of one-size-fits-all medicine, an effective treatment is the treatment known to benefit most of the target population, which is usually captured using the notion of *number needed to treat*. The number needed to treat (NNT) is a measure indicating the average number of people who need to be treated to avert one additional bad outcome. For instance, a commonly used treatment for cholesterol has a NNT of 20, which means 1 out of the 20 who are treated will actually yield benefit from the said treatment [7]. The rest of the population will not benefit from the treatment, and may even incur adverse effects. This exemplifies the need for customized treatment based on variables such as genetics, ethnicity or lifestyle. The underlying assumption is that precision medicine will provide tailored health care to patients and will yield lower rates of associated adverse outcomes. Although precision medicine aims at prevention, diagnosis and treatment, the main efforts have been centered around precision pharmacogenomics and the delivery of drugs based on patients’ specific genetic markers. For instance, the administration of drugs like clopidogrel is based on an individual’s genetic susceptibility for speedier metabolism [8] or risk for hypersensitivity to antiretroviral therapy abacavir is calculated based on a genetic test [9]. In the precision medicine paradigm, given detailed patient characteristics, it is possible to more accurately predict the expected effect of each treatment option and, thus, to optimize care.

### Are clinical trials precision medicine?

One may argue that clinical trials have always been operating with a precision medicine paradigm, by testing therapies on homogeneous set of patients who are most likely to benefit and yield the most favorable outcomes from the drug. However, even with randomization, participation in clinical trials is not uniform across demographic, social, genetic –excusing Mendelian randomization [10]– and other factors that influence health. Historically, women, minorities, children and pregnant women have many times been excluded or underrepresented in clinical trials, and although this landscape is changing, it has not yet reached the levels of representativeness of the general population [11, 12]. Therefore, clinical trials have been ‘precise’ only for a subset of the population. Additionally, the costs associated with sufficiently-powered clinical trials stratified across all possible outcome modifiers make them prohibitive as a cost-effective precision strategy.

### More variables, more observations

In order to be precise, medicine must revolve around data, especially in generating, linking, and learning from a variety of sources. This means going beyond genetics and exploring data that may not be traditionally thought of as being related to health and disease. However, resources need to be included as a key variable of precision medicine, regardless of the health system one considers. Indeed, health monitoring can quickly become expensive, and thus, cost-effective strategies need to be identified across the continuum of care. For instance, while there are biomarkers that are static in nature (e.g. a specific genetic variant), others change over time and need to be evaluated periodically. In an ideal world where a plethora of markers can be used to predict future health status with high precision, a cost-effective set should be identified in order to guarantee the same performance with minimal burden.

The main objective of this paper is to 1) explore the evolution of medicine and public health in a data-rich world; 2) present the current main hurdles to have precision health deliver on its promises; and subsequently 3) propose a modeling framework to remove some of these barriers to precision medicine and precision public health, improving health outcomes and reducing health disparities. To help the reader throughout the sections, we have prepared a summary list of the arguments of our debate, listed in Table 1.

**Table 1:** Hurdles in precision medicine and precision public health within data, study, model development, and deployment phases

Precision medicine			
• Concentration on individualized treatment and neglect of time component of predictions, i.e. early risk vs. differential diagnosis vs. post-treatment survival • Too much focus on genetics and –omics • Research on actionable factors vs. immutable risk factors • Integration of multi-omics • Integration of multi-domain data (e.g. genetics, diet, lifestyle, social)			
Precision public health			
• Definition of target units (e.g. ethnic groups, geographic zones, social groups) • Conflict with precision medicine, i.e. individual-centric objectives (benefit of the single may not translate into benefit of the population) • Population-level outcomes			
Data sources	Study designs	Prediction modelling	Translational relevance
• Heterogeneous data sources • Unstructured data sources • Lack of data on social determinants of health • Measurement issues (e.g. incompleteness, inaccuracy, imprecision in self-reported data) • Privacy and security • Cost • Limited adoption of common data models	• Semantic data integration (i.e. linking data elements by their meaning) • Large longitudinal cohorts • Ontology integration • Ontology appropriateness (e.g. ontologies made for billing vs. for diagnostic purposes) • Semantic interoperability • Automated study design	• Biases of all sorts (e.g. protopathic) • Confounding • Causal inference • Black-boxes vs. white-boxes (i.e. interpretability vs. performance) • Complexity-based model selection • Benchmark development • Pragmatic interoperability (reproducibility, replicability, generalizability)	• Limited individual empowerment • Disconnect from relevant clinical research • Personal health record/health avatar (besides provider’s electronic records) • Acceptance of artificial intelligence as integral part of doctors’ tools • Learning systems • Ethical usage and dissemination of modelling algorithms • Redefining disease phenotype

## The evolution of precision medicine

### Redefining precision care

**Fig. 1:**
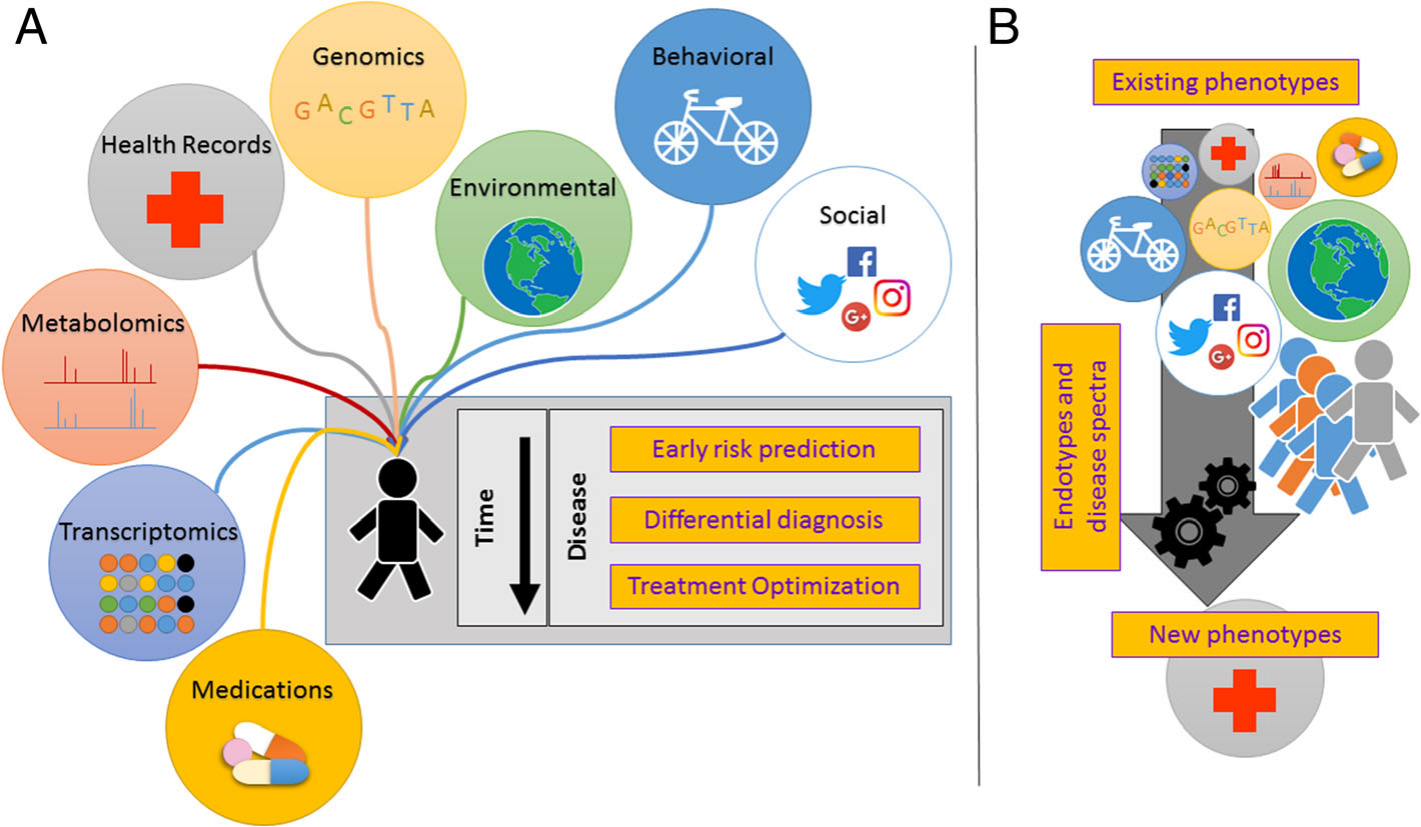
Revisitation of precision medicine. **a** Precision medicine moves from a genetics-centered personalization of treatment on to a dynamic risk assessment and optimization of current and future health status through immutable (e.g. genetics) and actionable factors (e.g. behavior). **b** Disease phenotypes are reclassified on the basis of new system-level evidence, identifying pathophysiological endotypes associated with common, known phenotypes. The logos are trademarks of their respective companies and institutions, and their uses do not represent affiliation or endorsement

Individualized treatment, e.g. tailored pharmacotherapy, is not the sole component of precision medicine. From a utilitarian point of view, it may be useful to break down precision medicine by its components across the continuum of care (Fig. 1a), to be met under specific time constraints:

- Disease prevention, or prediction of disease risk before the disease symptoms manifest,
- Differential diagnosis, or timely/instantaneous identification of an illness, and
- Disease treatment, i.e. strategies to cure or optimally treat once disease has been identified.

These components reflect the move from a focus on treatment only in health care to include also prevention, as well as prognosis, and post-disease survivorship, as critical aspects of medicine and health in general.

In the context of disease prevention, the goal of precision medicine is accurate prediction of disease risk –even years in advance. For instance, many risk factors associated with lung cancer are well understood, and even though behavior change is difficult, the timeframe is sufficient to intervene in order to decrease risks. For all diseases with potentially severe health outcomes, whose etiology is not entirely understood, prediction modelling should be used to identify disease markers as early as possible. However, prediction models are only useful if they can include risk factors that are modifiable –such as dietary habits and lifestyle (because genes, age, race are not). Such models would allow changing the odds of the disease onset, and possibly with enough time for an intervention. In many modelling approaches, as we will see in the next sections, the value of the data from the modifiability point of view is not taken in the account, as well as from a ‘comprehensibility’ point of view (i.e. understanding the mechanics of the underlying biological processes by decomposing the model functions).

With differential diagnosis, the timeframe is reduced to a matter of days or even hours. Acute abdominal pain can have very different etiology, ranging from aortic aneurysm to kidney stones, or peritonitis. Mistaking a chronic condition, e.g. Meniere’s, can severely affect quality of life [13]. Besides genetic markers, in this case we can think of high-sensitivity tests with rapid turnaround time, like metagenomics sequencing to screen for multiple pathogens. Many diseases and diagnoses are still not clearly defined: *“Descriptions of disease phenotypes often fail to capture the diverse manifestations of common diseases or to define subclasses of those diseases that predict the outcome or response to treatment. Phenotype descriptions are typically sloppy or imprecise”* [14, 15]. For instance, asthma is an umbrella disease, with possibly different underlying endotypes [16]. Rheumatoid arthritis and its related gamut of symptoms, or other types of autoimmune conditions, as well as Alzheimer’s disease are considered system-level illnesses [17]. Therefore, one additional constituent of precision medicine is the notion of ‘precise phenotyping’ (Fig. 1b).

The last point, i.e. disease treatment, is the last stand against unfavorable health outcomes. It necessarily builds upon the former two and moves forward by adding more complexity, i.e. the space of treatments. Different outcomes can be obtained by running prediction models that set a patient’s status (e.g. genes, metabolic profile, drug exposures) and vary treatments (e.g. new drugs, diet, physical activity). Treatment optimization, seen as an operational research problem, explores this outcome prediction space looking for the most favorable ones.

### Genetic epidemiology: the big short

The widespread availability of sequencing methods along with a drastic reduction of their associated costs were largely responsible for the rise and evolution of precision medicine. Today, genome-wide sequencing can cost about $1000, down from close to $98 M in 2001. Moreover, several commercial companies are offering services that provide partial genome sequencing for a little over $100, along with mapping to demographic traits and specific disease conditions (yet with unproven clinical utility), which isn’t without raising concerns related to privacy of health information [18]. Although a genome stays relatively immutable during a lifetime, a genomic screening obtained at birth or before birth will be optimal for the most accurate disease prediction [19]. Despite the initial enthusiasm for genetics-focused precision medicine, the results have been underwhelming and have not delivered on its promises so far. The predictive ability and ensuing clinical utility of risk assessment from genetic variations has been found to be modest for many diseases [20, 21], and genome-wide association studies (GWAS) have not led to understanding genetic mechanisms underlying the development of many diseases [22].

Among the shortcomings of GWAS, one is the *missing heritability* problem –heritability is a measure of the proportion of phenotypic variation between people explained by genetic variation– for which single genetic variations cannot account for much of the heritability of diseases, behaviors, and other phenotypes.

Another limitation of GWAS relates to studying a single phenotype or outcome (often imprecise, as we pointed out in the previous section), and accounting for heterogeneous phenotypes would require studies massive in size [23]. Other GWAS issues include design, power, failure of replication, and statistical limitations. In practice, only univariate and multivariable linear regression is performed. Looking at gene-gene interactions, and including other variables rather than basic demographics or clinical traits is rarely done and often computationally burdensome.

There are very few examples of high-effects common genetic variants influencing highly-prevalent diseases, and common genetic variants usually have low predictive ability. The rarer a genetic variant, the harder it is to power a study and ascertain the effect size. There are rare high-effect alleles causing Mendelian diseases and a glut of low-frequency variants with intermediate effects. Low-effect rare variants are very difficult to find and may be clinically irrelevant, unless implicated in more complex pathways [24]. In fact, it is known that gene expression pairs can jointly correlate with a disease phenotype, and higher-order interactions likely play a role too [25–27]. Few algorithms have been proposed to seek jointly-expressed genes, and existing methods are computationally inefficient [28].

However, these issues are only partially responsible for precision medicine not yet meeting its original promises. Indeed, for precision medicine and precision public health models to be valid and effective, incorporating and testing factors beyond genetics is key. While genetics remains mostly static over time, other health-related factors are constantly changing and need to be evaluated periodically. Epigenetics, e.g. methylation data, which has a time component, can contribute to a relevant portion of unexplained heritability [29]. Cheaper and faster production of sequence data with next-generation sequencing technologies has opened the post-GWAS era, allowing for a whole new world of –omics [30, 31].

### Domain-wide association studies

The GWAS revolution, and arguably saturation, has brought a surfeit of epigenome-wide –methylation-wide, transcriptome-wide– [32], microbiome-wide [33], and environment-wide association studies [34]. Interestingly, phenome-wide association studies reverse the canon, as all health conditions found in medical histories are used as variables and associated to single genetic traits [35].

Genomics, transcriptomics, metabolomics, and all other –omics can be seen as input *domains* to a prediction model. Merging of two or more domain-wide association studies is the next step toward a better characterization of disease mechanics and risks [36].

However, modelling and computational challenges arise with multi-domain integration, because of increased dimensions, variable heterogeneity, confounding, and causality. Formalizations of cross-domain-wide association studies, under the general umbrella term of *multiomics*, have been proposed [37–39]. Despite the cheaper and faster production of sequence data, most of the multiomics studies are limited by small samples: in general, the more heterogeneous the experimental data to be generated or the data sources to be included in the study are, the more difficult it is to get larger sample size.

The most interesting utility of multiomics, rather than prediction of health outcomes, is their ‘unsupervised’ analysis, i.e. the identification of patterns/endotypes that can help unveiling biological pathways, and eventually redefine disease spectra and phenotypes. However, there is mounting evidence that to ensure that precision medicine and precision public health deliver on their promises across the care continuum, we need to go beyond the –omics.

### Beyond traditional domains

In the era of precision medicine, multi-domain studies need to extend beyond ‘omics’ data and consider other domains in a person’s life. Specifically, genetic, behavioral, social, environmental, and clinical domains of life are thought to be the five domains that influence health [40]. Further, the ubiquitous nature of Internet access and the widespread availability and use of smartphone technologies suggest that the clinical domain can be significantly enhanced with patient-generated data, such as physical activity data, dietary intake, blood glucose, blood pressure, and other similar variables that can be seamlessly collected using smartphones and wearable devices [41].

Moreover, such tools, combined with social networks platforms provide a window into the behavioral and social domains of health, data-rich environments that need to be considered in the context of precision medicine and precision public health, to create a ‘digital phenotype’ of disease [42]. For instance, images from Instagram have been used to ascertain dietary habits [43] in lieu of a food diary or dietary intake questionnaires, which can be inaccurate, and are cumbersome and time-consuming; Instagram, again, has been used to identify predictive markers of depression [44]. The passively collected data from Twitter can be used for insomnia types characterization and prediction [42]. Research into the environmental domain has shown that the environment in which we live in impacts our health and mortality [45]. However, research using non-traditional health-related data from these domains have been conducted with some success as well as with some controversy [46, 47].

### The health avatar

**Fig. 2:**
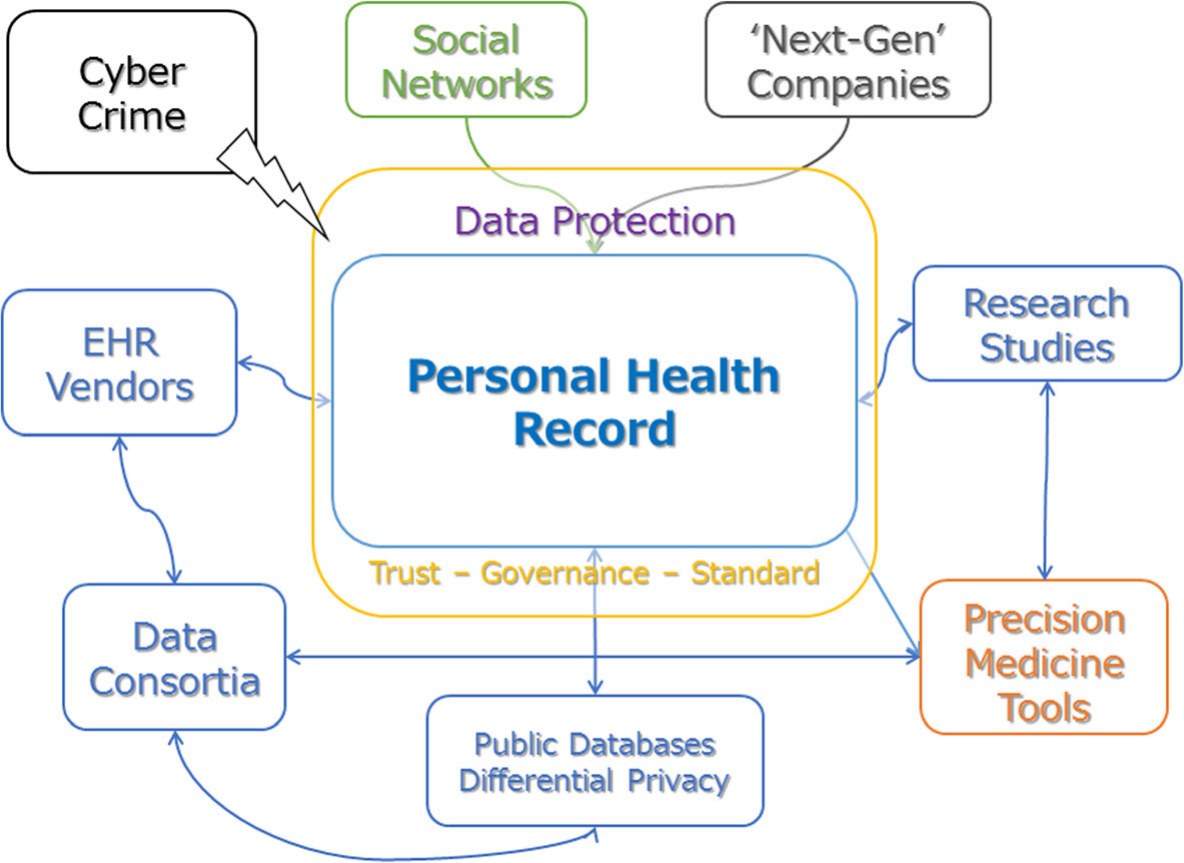
The health avatar: a virtual representation of a person with all their associated health information, and intelligent ways to manage and predict their future health status. The health avatar is centered on the personal health records and integrated with healthcare, commercial governance, and research entities

As precision medicine fundamentally reduces to fine-grained, individual-centric data mining, the observational unit of such data, and pivot for domain linkage, can be defined with the theoretical model of the *health avatar*. The health avatar is a virtual representation of a person with all their associated health information (Fig. 2), and intelligent ways to manage and predict their future health status [48].

Even with the widespread use of electronic health records (EHR) and integrated data repositories, individuals are generally detached from their health information and opportunities to be actively involved in research remain limited, despite initiatives such as Apple’s HealthKit. Well-known barriers to linking and efficiently exploiting health information across different sites slow down healthcare research and the development of individualized care. Further, EHR are not translationally integrated with diagnostic or treatment optimization tools. A doctor can get and transfer lab results online, but then diagnoses are often made in a traditional manner, based on average population data.

The personal health record (PHR) is a collation of all health information from different healthcare providers or other sources that is stored in the cloud, a directly accessible property of the individual [49, 50]. The PHR is complementary to the EHR, which is usually stored at the provider level, with vendors’ software, such as Epic [51] or Cerner [52]. However, the health avatar should not simplistically be identified with the PHR, as the PHR is inherently passive, with little involvement from the patient. We now propose a model of what the modern health avatar should be, in an era of large patient-generated data sets. An individual can see their health information using a provider’s PHR, but cannot easily merge the information with data from other providers nor ask a provider to upload their data from the EHR to the PHR simply during a doctor’s visit, e.g. via a smartphone app. An intelligent algorithm that matches people to research studies based on their full medical history does not exist yet. Both doctors and patients who are interested in computer-aided diagnosis, usually have to upload information to a third-party service (e.g. to analyze susceptibility to antibiotics). Finally, data shares are cumbersome, not only in terms of steps required to respect ethical principles, practice, and to protect human subjects, which are necessary but could be modernized, but also because the only data considered reliable are those coming from EHR. This means that big data shares happen solely at the population level via institutional or corporations’ liaise. Research and analytics that follow are not streamlined; the long-awaited *research objects* –semantically rich aggregations of resources that bring together data, methods and people in scientific investigations– are still in their infancy [53]. Integration of different types and sources of data should retain original context and meaning while meaningfully mapping their relationships to other health-related variables; such semantic integration will need to be flexible and comprehensive.

Physical data integration of EHRs requires enormous efforts and resources, but currently is the most successful approach to health information linkage because it is supported by rigorous governance standards and solid infrastructure. Efforts like the national patient-centered clinical research network [54] is a prominent example. Data sharing for matching research participants, one of the long-awaited prerogatives of NIH, is finally being exploited, via ResearchMatch [55].

The health avatar should link all and new types of health-related data, from genomics, to the myriad of -omics, mobile and wearable technology-based, and environmental sources. These data capture information from other domains which impact health far greater than clinical care alone. Such integrations have already begun around the world with healthcare systems like Geisinger conducting genetic sequencing and returning some of the results to the patients and with initiatives such as electronic Medical Records and Genomics (eMERGE) and Implementing Genomics in Practice (IGNITE) networks [56]. However, these efforts have been limited to genomics. More generally, the health avatar should be able to connect with and exploit non-EHR information potentially useful for health assessment, even coming from highly unstructured sources, such as social media. This is exemplified recently with Epic partnering with Apple to allow Apple’s HealthKit to display patient’s EHR data. Epic’s App Orchard also allows the collection of wearable technology data and storage into the EHR. For instance, an artificial intelligence tool could process images from Instagram and Facebook/Twitter posts to ascertain dietary habits, and this information can then be used to populate a food questionnaire, encoded into some type of structured information and stored in the EHR. Moving out of the individual level, environment-level information pertinent to the individual –for instance through residence ascertainment or mobile geolocation– could also populate EHR fields, storing information such as exposure to allergens and pollutants.

However, the collation of non-standard data, e.g. momentary ecological assessment via Twitter, Facebook, or smartphone GPS monitoring, is prone to serious privacy and security concerns. Ubiquitous approaches must be foreseen, as in the *Internet of things* [57, 58]. Data integration, and even more data share, must be secure to meet popular support. In this sense, the research in *differential privacy* aims at developing new algorithms not only to protect identities, but also to generate masked or synthetic data that can be shared publicly and freely used for preliminary research [59–61]. While differential privacy has facilitated data sharing, it remains challenging to safely anonymize data while preserving all their multivariate statistical properties [62]. The individual-centric approach of the health avatar can facilitate the match of individuals with research programs, with blurred boundaries between clinical care and research, while respecting ethics but modernizing informed consent concepts.

In terms of active features, i.e. not only data storage, the health avatar would feature linkage to personalized predictive tools for health status. Within the context of appropriate ethics bylaws and informed consents, health avatars could directly feed individual-level health information to multiple research projects for creating new and more accurate precision medicine tools. This would require data privacy and protection measures to avoid identity or data theft and misuse. Further, wide access to patient-generated data, along with integration with clinical and health databases provide a unique opportunity to expand precision medicine to the population level. We discuss this specific expansion in the following section.

### Precision public health

The Director of Office of Public Health Genomics at the Centers for Diseases Control and Prevention (CDC) defined ‘precision’ in the context of public health as *“improving the ability to prevent disease, promote health, and reduce health disparities in populations by: 1) applying emerging methods and technologies for measuring disease, pathogens, exposures, behaviors, and susceptibility in populations; 2) developing policies and targeted implementation programs to improve health”* [63]. Top priorities included: early detection of outbreaks, modernizing surveillance, and targeted health interventions. To achieve such improvements, comprehensive and real-time data to learn from are necessary. Epidemiology must expand surveillance on to multiple, different information domains, such as the Internet and social media, e.g. infodemiology [64]. Big data does not only mean large sample size or fine-grained sampling, but also large variety of variables. So far, the big data emphasis is on sequencing genomes population-wide [65, 66], but research has started to consider other domains, such as in integrating classical surveillance with geospatial modelling [67].

In yet another step-by-step guide to precision public health –focused on developing countries– better surveillance data, better data analyses, and rapid actions are urged: again, big data is the key, with emphasis on public data sharing, and on the data attributes of speed-accuracy-equity. Notably, this is an epidemiological projection of the canonical big data characteristics, known as the Vs [68, 69].

Winston Churchill famously stated that *“healthy citizens are the greatest asset any country can have”*, and to achieve health for all citizens, there needs to be a transition from precision medicine, which is individualized, to precision public health. In fact, precision medicine can be used to improve an individual’s health, but this does not necessarily translate into a uniform benefit for the population [70]. For instance, a precision medicine model tuned for majority of a population may improve the average health outcomes overall yet neglect minorities. To some extent, the term precision put next to population-wise priorities seems conflictual, and this may be due to application of single precision public health model to an entire population, rather than use of multiple segmented/cluster level models. Another fuzzy aspect of current precision public health approach is the lack of consensus on observational units used for inference or intervention [71]. Is it the individual? Is it a common geographic area? Is it a particular subpopulation? A theory-based approach in this sense would be useful, as we will show in the next section.

**Fig. 3:**
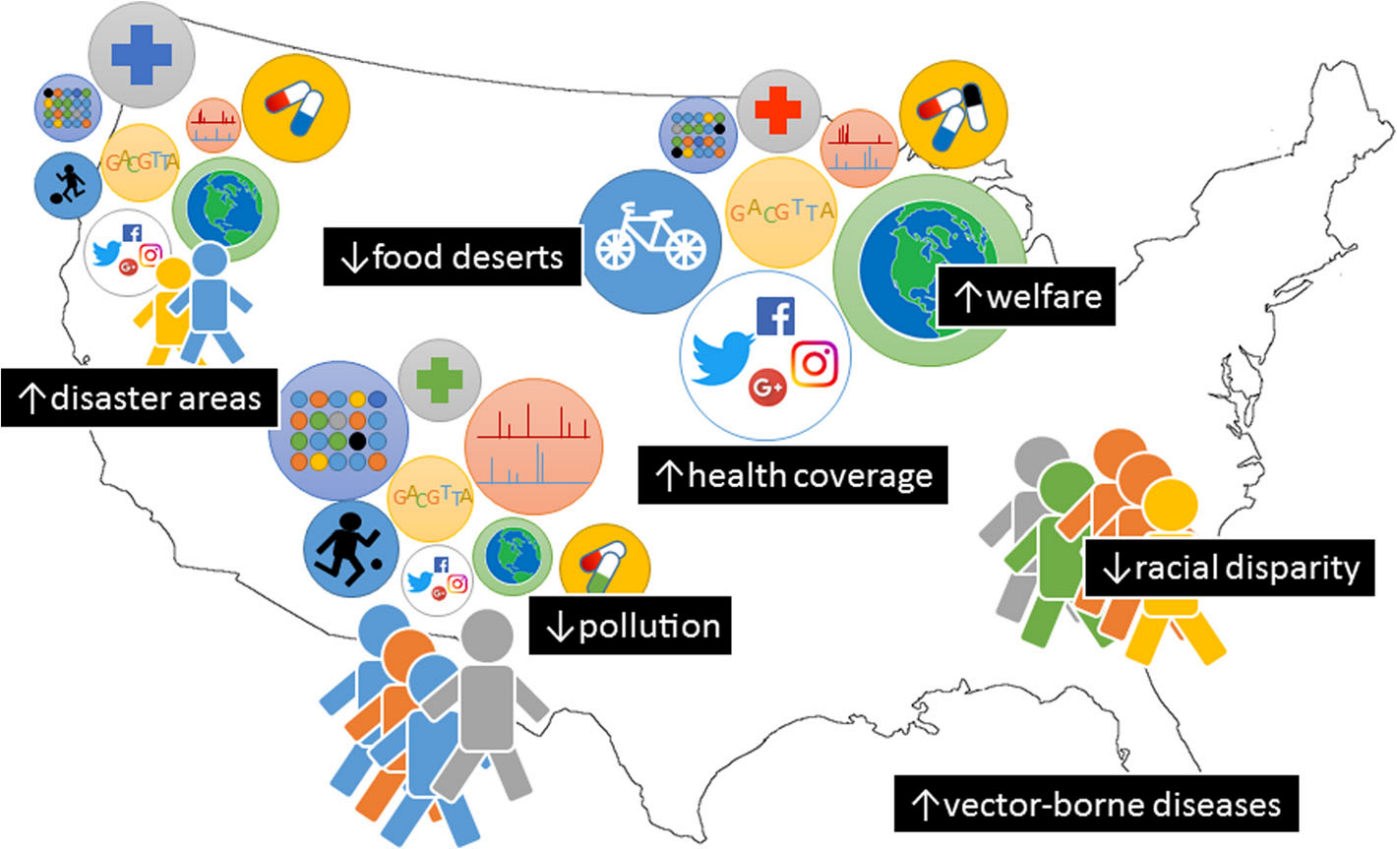
Precision public health. Community, societal and ecological factors must be accounted on top of the individual-based, fine-grained approach for precision medicine. The map is an edited version of a Wikimedia Commons image (https://commons.wikimedia.org/wiki/File:United_States_Administrative_Divisions_Blank.png, licensed under the Creative Commons Attribution-Share Alike 3.0 Unported)

Precision public health has to face societal challenges, including racial disparities (both in terms of welfare and genetic background), environmental niches (e.g. tropical climate with higher rates of arboviral diseases, industrial areas with high pollution), and general ethical concerns (religious beliefs, political views). An individual-centric model such as the health avatar here poses a number of limitations, because it may lack of higher-level dynamics happening at the societal-environmental level (Fig. 3). Interestingly, such dynamics can also influence the individual itself, and therefore should be accounted for and projected on to the person-centric models.

## Big data modelling for precision medicine and precision public health

### Semantic integration

Barriers to linking and efficiently exploiting health information across different sites slow down healthcare research and the development of individualized care. Different EHR systems may independently define their own data structural formats. This independent and heterogeneous management poses challenges in information mapping and encoding, e.g. merging data from multiple EHR systems or from different standardization procedures without access to the original data.

Data integration across multiple domains and sources is a daunting task due to at least three factors: 1) the heterogeneity in the syntax of the data such as the different file formats and access protocols used, 2) multiple schema or data structures, and more importantly 3) the different or ambiguous semantics (e.g. meanings or interpretations). Substantial effort is required to link different sources due to lack of clear semantic definitions of variables, measures, and constructs, but it can be eased by *semantic interoperability,* which allows exchange of data with unambiguous, shared meaning [72–74].

A common approach in semantic data integration is through the use of *ontologies.* Building upon a standardized and controlled vocabulary for describing data elements and the relationships between the elements, an ontology can formally and computationally represents a domain of knowledge [75, 76]. With a universal conceptual representation of all the information across different sources, a semantic integration approach allows us to bridge the heterogeneity of data across multiple sources and domains. Many biomedical ontologies are already available and widely used in medicine, e.g. the International Classification of Diseases (ICD) or the Systematized Nomenclature of Medicine and Clinical Terms (SNOMED CT) [77, 78]. Nevertheless, an unified ontology-driven data integration framework is needed to accommodate the growing needs of linking and integrating data from multiple domains. Going beyond traditional approaches of using *common data elements* and *common data models (CDM)* [79], such as the international efforts in building the Observational Medical Outcomes Partnership (OMOP) CDM [80], an ontology-driven data integration framework can be used to represent metadata, create global concept maps, automate data quality checks, and support high-level semantic queries. Further, research on the semantics of EHR improves not only data integration and interoperability, but can also advance the science on disease phenotyping [81–84].

Moreover, ontologies can be used to facilitate a formal documentation of the data integration processes (e.g. through encoding the relationships between the variables to be integrated across different sources). Doing so can have significant impact on research rigor, transparency, and reproducibility among scientists as well as data reusability and flexibility.

**Fig. 4:**
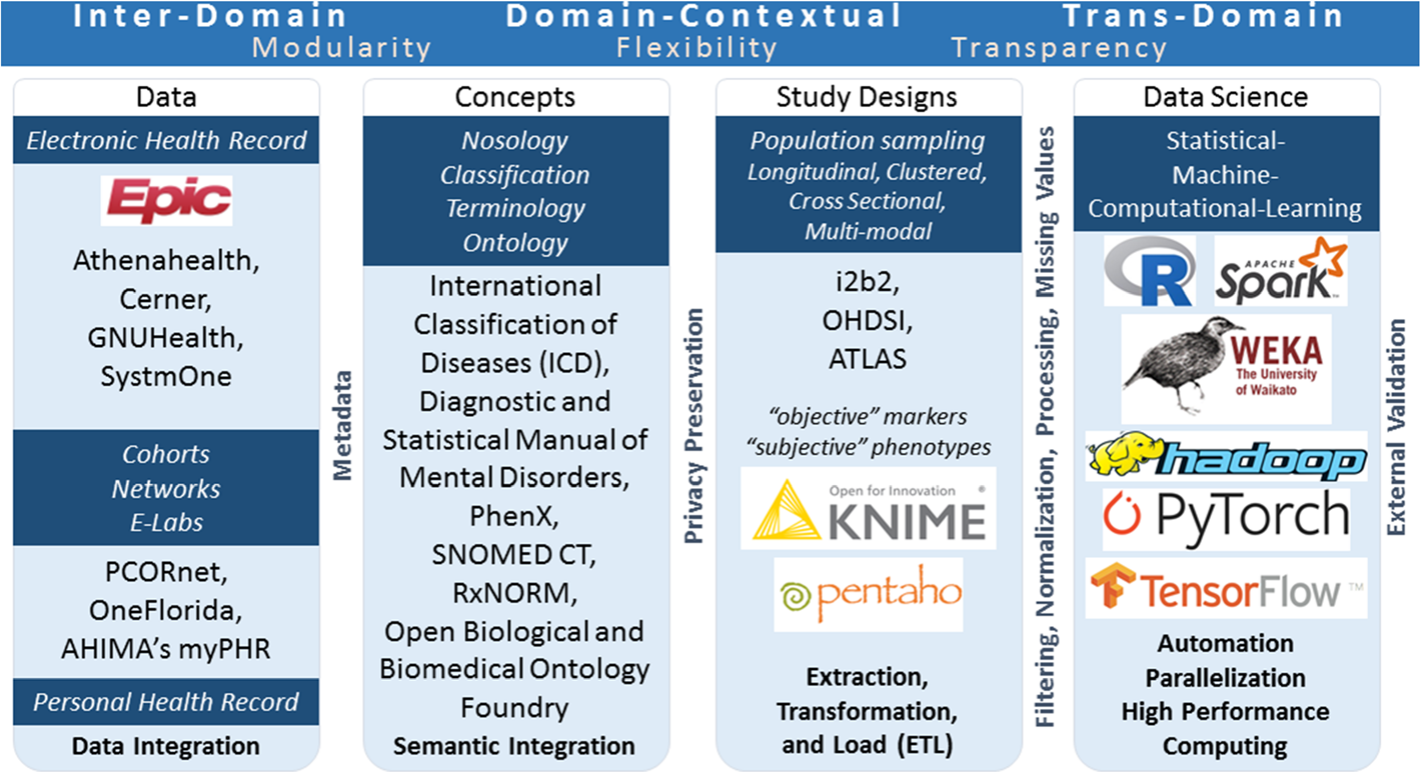
Semantic integration on data, study design and inference. The logos are trademarks of their respective companies and institutions, and their uses do not represent affiliation or endorsement. TensorFlow, the TensorFlow logo and any related marks are trademarks of Google Inc. The logos are used for informative purposes only, and the list included here is not exhaustive

Semantic integration can occur at different levels of healthcare research, not only at the data level with EHR. As mentioned, study designs on integrated data sources need to be supported by proper semantics. In Fig. 4 we summarize the semantic integration paradigm at different levels: (i) the data level, integrating both EHR and PHR data sources (inter-domain); (ii) the concept level, mapping terminologies and ontologies (domain-contextual); (iii) the study design level, enabling standard operating procedures and reproducibility on other sources (domain-contextual); (iv) the inference level, identifying proper statistical learning methods upon study design, scaling analyses on high-performance computing, and building up models and applications for the public health benefit (trans-domain). Semantic integration allows modularity (e.g. addition of new data or ontology components), flexibility (e.g. modification of existing study designs or execution in different environments), and transparency (e.g. reproducibility of results, validation, enhancement of models).

For instance, interoperable semantics and research objects have been the driver to the ‘asthma e-lab’ project [85]. As a secure web-based environment to support data integration, description and sharing, the e-lab is coupled with computational resources and a scientific social network to support collaborative research and knowledge transfer.

Another relevant example is the Observational Health Data Sciences and Informatics (OHDSI) [86] consortium, whose goal is *“to create and apply open-source data analytic solutions to a large network of health databases to improve human health and wellbeing.”* OHDSI uses the OMOP common data model and features a suite of applications for streamlining integration of EHR, data quality assessment and cleaning (ACHILLES), standardized vocabulary for OMOP (ATHENA), data query and cohort identification (ATLAS), and analytics (CYCLOPS).

With semantic interoperability standing, we move on to study design theorization for precision medicine and precision public health.

### Study designs: hollow learning, shallow design

With the advancements in technology and data linkage, single-domain research is being superseded by multi-level, multi-domain studies. Such increase in complexity and heterogeneity of studies affects also their design, in the case of both prospective and observational designs. Especially for observational studies, there is huge amount of data potentially available, but the access and use of such heterogeneous data sources must be rationalized to tackle bias, identify actionable inputs, and consider ethical needs.

**Fig. 5:**
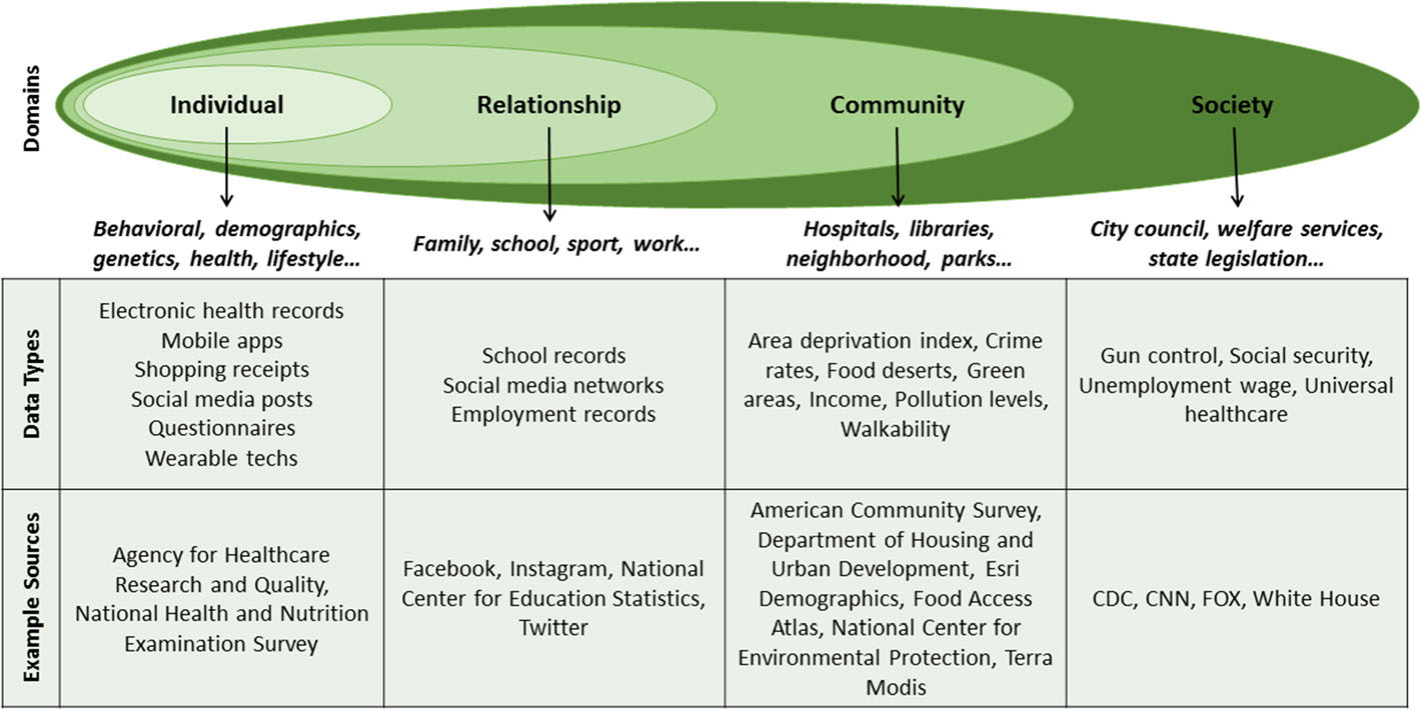
The social-ecological model with associated information domains and data sources for a multi-domain study design

In psychology research, it has been proposed that data-driven studies should be guided by an etiological theory in terms of study design [87]. These theories are grounded on evaluating scientific evidence as causal pathways of disease. Hybridization of using theory to guide design (‘top-down’ approach) with data-driven research (‘bottom-up’ approach) can be very useful for development of multi-level and multi-domain prediction models, encompassing individual and population levels. Several conceptual models exist that can be used, such as the social-ecological model or the multi-causality model [88, 89]. The challenge when using such models is to identify the sources of information for each component and to link the data, as we just discussed in the health avatar and semantic integration sections. In Fig. 5, we show the social-ecological model, the information domains, and a number of data sources (mostly available in the United States, for illustrative purposes) that can be used to extract relevant attributes for the domain dimensions.

The advantage of using a theoretical model is that it is possible to deconstruct the prediction model to test hypotheses or identify new areas that need further investigation. For example, suppose we use the social-ecological model and integrate individual-level EHR and genetic markers with community-level social and ecological indicators, over a specific time horizon, to determine population risk of acute or chronic asthma. Certain variables in the individual- or the community-levels may be found to contribute to increased risk, and through cross-domain interactions, the percentage of variance explained may increase. Furthermore, variables in each domain can be examined to see if they are actionable or immutable (e.g. environmental exposures vs. genetics) and ethically usable or not (e.g. neighborhood deprivation score vs. racial profiling). This information can be exploited to determine a proper risk model and to select factors that can be modified to reduce the risk of disease.

One of the biggest hurdles in study design, especially for observational or retrospective studies, relates to effectively identifying and addressing bias. With big data, this issue is severe, because of data collection heterogeneity, source verification, and sampling bias among others. Researchers must be wary of the ‘big data hubris’ or *“that big data are a substitute for, rather than a supplement to, traditional data collection and analysis”* [46]. With EHR, bias overwhelms randomization. EHR data are inherently biased by the patient population structure, frequency of health care visits, diagnostic criteria, and care pathways; drug prescription records mostly reflect indication or protopathic bias. Even the most advanced statistical methods cannot disentangle bias, but they can learn it very precisely. Therefore, feeding a deep learning architecture with raw EHR data may be a very bad idea, although it yields amazing prediction performance [90–93]. In fact, *“biased algorithms are everywhere, and no one seems to care”* [94]. The problem is not novel and becomes dangerous if used for decision making [95]. Besides tragicomic revamping of phrenology through deep learning [96], ProPublica’s assessment of the Correctional Offender Management Profiling for Alternative Sanctions (COMPAS) algorithm, a tool used to predict a person’s risk of recidivism, is a serious example of bias-learning models [97].

### Prediction modelling: interpretability vs. performance

**Fig. 6:**
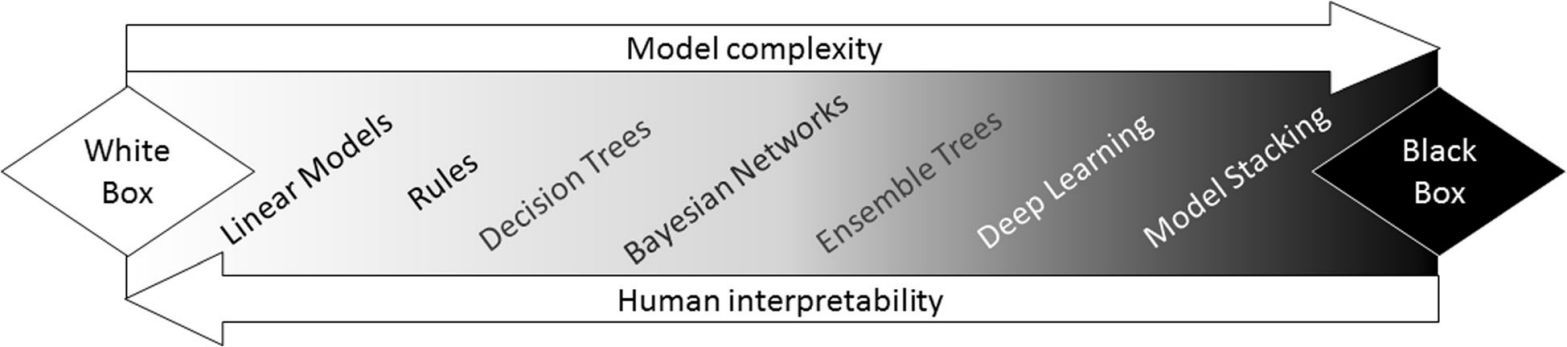
Machine learning models: white- and black-boxes. Increasing model complexity can lead to better approximation of functions and enhance prediction performance, but can lead to a decrease in interpretability of the model

Another important challenge in use of big data for precision public health is the utility of inferred models, i.e. *“Do big data lead to big models?”* ‘Big’ models contain many variables and in nonlinear or highly complex ways, and such machine learning models can yield easily interpretable results or excellent prediction, but not necessarily both at the same time. In spite of the potentially higher accuracy in predicting disease diagnoses and health outcomes, many machine learning methods are usually regarded as non-transparent to the end user and labeled as *black-boxes.* In opposition, *white-boxes* are human-interpretable models, such as risk scores or diagnostic rules. Although black-box models may provide a very precise calculation of the probability of a target event or outcome, they are often regarded with skepticism due to the lack of consideration for causal pathways (Fig. 6). However, when integrated seamlessly in EHR as a clinical decisions support system and if they can identify clinically actionable features, they can be more acceptable [98].

**Fig. 7:**
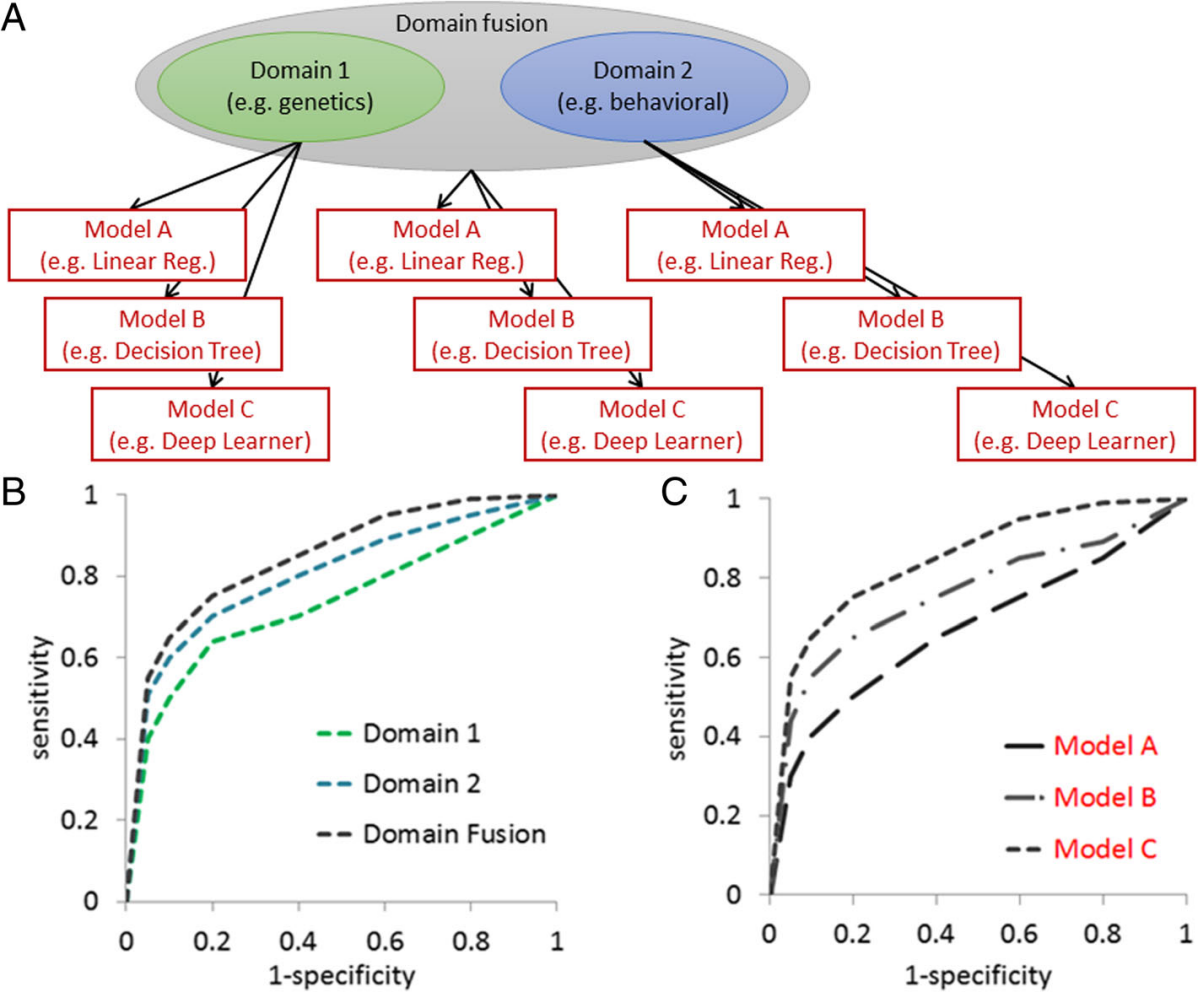
Domain-guided and complexity-guided model selection. **a** Hypothetical data set with two domains and their merged domain, on which models of increasing complexity (linear regression, decision tree, and deep learner) are fit and compared. For example, using the receiver-operating characteristic, (**b**) the predictive performance of a prime model can be assessed using a single domain or merged domains, or (**c**) different models can be compared within the same domain space

Management of the tradeoff between interpretability and prediction performance is often neglected when developing frameworks for predictive analytics, but it can be critical for deploying the models in clinical practice [99]. One possible way to balance between white- and black-boxes is to use the more complex strategy known as the *super learning* framework [100], or *stacking*, and deconstruct its components. Essentially, the super learning approach fits and stacks many different models together on the data and selects the best weighted combination. Although super learning approaches are thought to have maximal prediction accuracy and minimal interpretability, deconstruction into digestible components is a necessary step for interpretability and thus, clinical utility. This can be extended to test various domains to include in the model, to optimize the model, and to guide future explorations of the data (Fig. 7).

For instance, the super ICU learning algorithm (SICULA) has been constructed for mortality prediction [101]. Post-hoc tools to identify the importance of individual variables can break down the complexity of black-box models like the random forest or neural networks [102, 103].

Model complexity is not universally defined, but indices like the Vapnik-Chervonenkis dimension can be used [104]. When selecting models on the basis of their complexity, there are two advantages: 1) performance thresholds can be set on the basis of clinical utility such that a more interpretable model that is less accurate than a more complex one could be chosen if it meets the required sensitivity or specificity; and 2) model simplification and interpretation can help in understanding findings to develop new etiological or mechanistic hypotheses. Nonetheless, the picture is not as simple: there is no guarantee that the combined information induced by a super learner will be straightforward to deconstruct; if the final models are deep neural networks, they will still be very challenging to interpret. The interpretability of complex and/or stacked models will still be limited by the inherent interpretability of the underlying components and functions. Downstream analysis like variable importance ranking or partial dependence plots may be helpful, but these solutions are highly model-dependent and can be biased by numerous factors (such as variable collinearity).

### Modelling interoperability

Besides semantic interoperability, interoperability of the modelling phases is needed. Using the standardized levels of conceptual interoperability, modelling interoperability can be abstracted as *“pragmatic interoperability*,*”* i.e. methods’ and procedures’ awareness, lying above semantic interoperability [105]. Reps et al. recently introduced a standardized framework that leverages OHDSI and OMOP not only for *“transparently defining the problem and selecting suitable datasets,”* (i.e. semantics) but also for *“constructing variables from the observational data, learning the predictive model, and validating the model performance”* (i.e. modelling) [106].

### Translational relevance

For any precision public health model to be useful, it should be robust to noise and generalizable; they should also be transparently presented in terms of their performance and reproducibility [107], and software libraries for differential privacy should be enforced as generic templates to facilitate data sharing and reproducibility of the works. When utilizing these models, we must consider whether the findings go beyond statistical significance and signify realms of clinical relevance. As previously mentioned, identification of risk factors which are immutable are impractical for interventions, and in cases of diseases where there are no treatments, accuracy of disease diagnoses will not impact clinical treatment decisions; however, additional insight on the mechanics of disease progression may be gained.

Linkage and systematization of data across multiple domains of life has the potential to increase patient education and participation in health care [108]. This in turn could lead to improvement in patient empowerment and shared decision-making, which are associated with improved health outcomes. By creating an access point for individuals to view their EHR and other variables that may affect their health, the health avatar empowers patients to take action. Impact of such empowerment has shown to modify health behaviors to reduce the risk of rheumatoid arthritis [109] and to make preparations for ill health in the future [110]. Moreover, health avatars can be venues for increased visibility of available health care facilities and ease of connection to care; this is currently being tested with wearable technology that can detect atrial fibrillation and prompt connection to a physician through mobile devices [111]. In addition to the impact on physicians for clinical decision support and on patient empowerment, the health avatar can be the missing intelligent algorithm that matches people to research studies based on their full medical history and other health-related factors. This will allow researchers to reach populations in vast numbers and allow implementation of novel study designs, such as examining rare adverse effects of a drug which randomized clinical trials cannot be sufficiently powered to detect [112].

The landscape of public health is evolving to a multi-domain, multi-stakeholder undertaking. The Food and Drug Administration is piloting digital health software programs. Companies which are outside of the health care domain are now engaged in creating health care programs for their employees [113].

However, a number of basic hurdles still remain open: prediction models of future health statuses are not yet accurate, and their actionability, i.e. changing the odds that a disease will occur, is even less accounted for; precision public health lack of contextualization within a societal and ecological environment; and integration with ethics and policymaking. Finally, affordability, trust, and education of the masses to this new paradigm of medicine will need to be addressed soon.

## Conclusions

In this work, we have discussed the promises of precision medicine and precision public health, as well as the challenges we face to leverage big data for precision care that could lead to effective advancements and translational implementations. Thus, the aim of this paper was to provide a critical and objective view of where we are, and the work that needs to be done to achieve true precision medicine and precision public health, to improve health outcomes, and to reduce health disparities. In particular, we have revisited some of the definitions and described a hybrid theory-based and data-driven approach that can aid with the processes of study design and model inference. A hybrid approach allows us to tailor the modelling to specific problems and needs. The top-down approach relies on strong prior knowledge, which can be used to guide study design (e.g. domain selection, observational units, cohort identification) and test specific hypotheses (such as in clinical trials). On the other hand, the bottom-up approach helps in exploring a large variety of hypotheses with weaker assumptions.

Precision medicine demands interdisciplinary expertise that understands and bridges multiple disciplines and domains up to a point where the fulcrum of the research is located on the bridges themselves. This defines *transdisciplinarity*, knowledge discovery going beyond disciplines, which demands new research and development paradigms.
